# Antiamnesic Effect of *Actinidia arguta* Extract Intake in a Mouse Model of TMT-Induced Learning and Memory Dysfunction

**DOI:** 10.1155/2015/876484

**Published:** 2015-10-21

**Authors:** Jeong Su Ha, Dong Eun Jin, Seon Kyeong Park, Chang Hyeon Park, Tae Wan Seung, Dong-Won Bae, Dae-Ok Kim, Ho Jin Heo

**Affiliations:** ^1^Division of Applied Life Science, Institute of Agriculture and Life Science, Gyeongsang National University, Jinju 660-701, Republic of Korea; ^2^Center for Research Facilities, Gyeongsang National University, Jinju 660-701, Republic of Korea; ^3^Department of Food Science and Biotechnology, Kyung Hee University, Yongin 446-701, Republic of Korea

## Abstract

The antiamnesic effects of ethyl acetate fraction from *Actinidia arguta* (EFAA) on trimethyltin- (TMT-) induced memory impairment were investigated to find the possibility of functional food substances. EFAA showed a potent AChE inhibitory effect (IC_50_ = 53 *μ*g/mL) and efficient neuroprotection against H_2_O_2_-induced oxidative stress. The administration of EFAA significantly decreased TMT-induced cognitive deficit in Y-maze, passive avoidance, and Morris water maze (MWM) tests. After the behavioral tests, the antioxidant activities were confirmed using mice brain tissues. EFAA not only showed the inhibition of AChE activity and the decline of malondialdehyde (MDA) level as a sign of lipid peroxidation but also presented the increase of the superoxide dismutase (SOD) level and the decrease of the oxidized glutathione (GSSG)/total glutathione (GSH + GSSG) ratio. Finally, the phenolics in EFAA were identified using liquid chromatography coupled with hybrid triple quadrupole-linear ion trap mass spectrometry, and four main phenolics, such as quinic acid, chlorogenic acid, caffeoyl hexose, and quercetin-3-glucoside, were identified. These results suggest that EFAA containing physiological phenolics might enhance drug-induced amnesia through AChE inhibition and neuroprotection.

## 1. Introduction

Alzheimer's disease (AD), which is one of the most serious diseases in the aged societies of developed countries, is a neurodegenerative disorder characterized by loss of learning and memory. Cholinergic hypothesis was proposed as an AD pathogenesis, owing to its relation to the extensive loss of neurons in the nucleus basalis of Meynert [1]. A decrease of choline acetyltransferase (ChAT) levels and an increase of acetylcholinesterase (AChE) levels were related to cognitive dysfunction through the decrease of acetylcholine (ACh) levels, which is a neurotransmitter [2, 3].

Reactive oxygen species (ROS), the products of oxygen metabolic processes, such as the superoxide anion radical (^•^O_2_^−^), hydrogen peroxide (H_2_O_2_), and hydroxyl radical (^•^OH), can cause neuronal apoptosis and impair cellular function and membrane integrity [4]. Many researchers have not only demonstrated an increase of oxidative stress in the brains of AD patients but also reported that the increase of antioxidant uptake is inversely related to the risk of AD incidence [5]. Some phenolic compounds through the intake of foods such as fruits or vegetables may reduce the risk of AD owing to their antioxidant properties, which protect the neuronal cell from oxidative stress caused by ROS, which may be related to neurodegenerative disease [6]. Recently, phenolic compounds have been studied as a source of natural antioxidants [7, 8].

*Actinidia arguta* (*A. arguta*), which belongs to the family Actinidiaceae, is a high-value food resource, globally 2–5 genera, 280–560 species, and native* Actinidia* spp. in Korea include* A. arguta*,* A. polygama*,* A. rufa*, and* A. kolomikta* [9].* Actinidia* spp. in Korea have strong cold resistance and pest resistance, and the whole of the fruit can be eaten without peeling it, owing to its thin bark, hairless, and mouthful-sized properties. Although its size is relatively small compared with the kiwi, it can be utilized in a variety of foods owing to its high sugar content and various nutrients, including vitamin C.* A. arguta* is known to alleviate fever and thirst as well as dyspepsia in Korean folk medicine [10]. The root of* A. arguta* is also used for the treatment of vomiting and arthralgia, and its rich vitamin C content can prevent fatigue and scurvy.

Trimethyltin (TMT) is an organometal neurotoxic compound. TMT exposure in rats has been reported to induce extensive hippocampal damage as well as abnormal behavior, such as hyperactivity [11]. Additionally, behavioral tests using TMT-induced animals are useful for the study of memory dysfunction, such as neurodegenerative disease [12].

The physiological activities of* A. arguta*, including the antiallergic, anti-inflammatory, antidiabetic, and antioxidant effects, have been reported by several recent studies [13, 14]. However, research on* A. arguta* related to cognitive function is insufficient, and most of all, physiological and cognitive improvement effects have not yet been reported. Consequently, the aim of the present study is to evaluate ameliorating effect of* A. arguta* on TMT-induced learning and memory deficits in ICR mice and is to identify main phenolic compounds.

## 2. Materials and Methods

### 2.1. Materials

Vitamin C, thiobarbituric acid, acetylthiocholine, H_2_O_2_, TMT, dimethyl sulfoxide (DMSO), 2′,7′-dichlorofluorescein diacetate (DCF-DA), 2′,3-(4,5-dimethylthiazol-2-yl)-2,5-diphenyl tetrazolium bromide (MTT) assay kit, lactate dehydrogenase (LDH) assay kit, 9-amino-1,2,3,4-tetrahydroacridine hydrochloride hydrate (tacrine), superoxide dismutase (SOD) assay kit, and solvents were purchased from Sigma-Aldrich Chemical Co. (St. Louis, MO, USA), and glutathione (GSH) detection kit was also purchased from Enzo Life Science Inc. (Enzo Diagnostics, NY, USA).

### 2.2. Extraction of* A. arguta*

The fruit of* A. arguta* (cultivar: Autumn Sense) was received from the Korea Forest Research Institute in September 2013 and was authenticated by the Korea Forest Research Institute. A voucher specimen was deposited at the Herbarium of the Department of Special Purpose Trees, Korea Forest Research Institute. After* A. arguta* was washed with running tap water, it was ground. Mixture (200 g) was suspended and extracted with 80% ethanol (4 L) at 60°C for 2 h. The extracts were filtered through Whatman number 2 filter paper (Whatman International Limited, Kent, UK) and evaporated. The evaporated materials were redissolved until 300 mL of distilled water. These redissolved solutions were consecutively partitioned in a separatory funnel with the equivalent amount of three solvents (*n*-hexane, chloroform, and ethyl acetate). The fractions were concentrated in a vacuum evaporator (N-N series, EYELA Co., Tokyo, Japan) at 60°C and were lyophilized. The lyophilized ethyl acetate fraction from* A. arguta* (EFAA) was stored at −20°C until used.

### 2.3. AChE Inhibitory Assay

The AChE inhibitory activity was carried out by the Ellman method using acetylthiocholine iodide as a substrate [15]. Cultured fluid of PC 12 cells was homogenized with 5 mL lysis buffer (pH 7.4), containing 10 mM Tris-HCl, 1 M NaCl, 50 mM MgCl_2_, and 1% Triton X-100 using the Glas-Col homogenizer, and supernatant was obtained by centrifugation at 14,000 g for 30 min. The supernatant was used as an enzyme, and all processing was performed at 4°C. Protein level in the supernatant was measured using the Quant-iT protein assay kit (Invitrogen, Carlsbad, CA, USA). After adding each 10 *μ*L EFAA and 10 *μ*L enzymes, preincubation was at 37°C for 15 min. Then after adding an Ellman reaction mixture in a 50 mM sodium phosphate buffer (pH 8.0) to the above reaction mixture absorbance was measured at 405 nm.

### 2.4. Neuronal Cell Culture and Measurement of Intracellular Oxidative Stress

PC 12 cells (KCLB 21721; Korea Cell Line Bank, Seoul, Korea) were reproduced in an RPMI 1640 medium (Gibco BRL, Grand Island, NY, USA) containing 10% fetal bovine serum, 25 mM HEPES, 25 mM sodium bicarbonate, 50 units/mL penicillin, and 100 *μ*g/mL streptomycin. The cells were cultured under the conditions (5% CO_2_ and 37°C).

Levels of intracellular ROS were measured using the DCF-DA assay [4]. DCF-DA as a nonfluorescent compound, upon entering into a cell, is deesterified, and then it forms substrate (fluorescence DCF) by intracellular ROS such as H_2_O_2_. Cells (10^4^ cells/well on 96-well plate) were treated with EFAA or vitamin C (positive control). After 48 h, cells were treated with or without 200 *μ*M H_2_O_2_, and cells were incubated for 2 h. Finally, cells were treated by the 50 *μ*M DCF-DA dissolved in phosphate buffered saline (PBS). Fluorescence was measured by fluorescence microplate reader (Infinite 200, Tecan Co., San Jose, CA, USA) with 485 nm excitation and 530 nm emission filters.

### 2.5. Determination of Cell Viability

Neuroprotective effect on H_2_O_2_-induced oxidative stress was measured by the MTT reduction assay. PC 12 cells (10^4^ cells/well on 96-well) were treated with EFAA or vitamin C (positive control); then they were preincubated for 48 h. The cells were treated with or without 200 *μ*M H_2_O_2_ for 3 h. The amount of formed MTT formazan by ability to return of mitochondria in living cells was measured using a microplate reader (Bio-Rad, Tokyo, Japan) at a test wavelength of 570 nm and a reference wavelength of 690 nm [4].

Protective effect of neuronal cell membrane was also confirmed using the LDH assay kit. In brief, cells were settled by centrifugation at 250 g for 4 min, and 100 *μ*L of supernatants was transferred into new 96-well. Damage of neuronal cell membrane was evaluated by measuring the amount of the intracellular enzyme, LDH released into the medium [4].

### 2.6. Animals

All experimental procedures were approved by the guidelines established by the Animal Care and Use Committee of Gyeongsang National University (certificate: GNU-131105-M0067). The Institute of Cancer Research (ICR) mice (4 weeks old, male) were purchased from Samtako (Osan, Korea), and mice were housed two per cage in a room maintained with a 12 h light-dark cycle, 55% humidity, and 23–25°C temperature. The EFAA was dissolved in drinking water at concentrations of 5, 10, and 20 mg/kg body weight, and once a day was oral administration through stomach tube. The mice were allowed free access to feed and water for 3 weeks. After 3 weeks, the TMT (7.1 *μ*g/kg of body weight (2.5 mg/kg)) was dissolved in 0.85% sodium chloride solution (w/v), and mice were intraperitoneally treated with a single injection (100 *μ*L). The control group was injected (100 *μ*L) with only sodium chloride solution without TMT [16].

### 2.7. Behavioral Tests in TMT-Induced Amnesic Mouse Model

Recording spontaneous alternation behavior in a Y-maze test was used to evaluate the immediate spatial working memory performance with the SMART video-tracking system (SMART v3.0, Panlab SL, Barcelona, Spain). The Y-maze test was performed after 3 days on the TMT injection. The maze was made of black-painted plastic, and each arm of the maze was 33 cm long, 15 cm high, and 10 cm wide and was positioned at equal angle. Each mouse was placed at the end of one arm and allowed to move freely through the maze for 8 min. The arm entry of mouse was considered to have been completed only when the hind paws of the mouse were placed completely in the arm of the maze. Alternation behavior is defined as successive entries into the three arms in an overlapping triplet set. The percentage of alternation behavior was calculated by the following formula using the total arm entry with a score [17]:

(1) Alternation  behavior%=Actual  alternationMaximum  alternation×100,Maximum  alternation=total  number  of  arm  entries−2.

The passive avoidance test was performed to investigate the short-term memory ability. Test box was divided into two parts, one illuminated and one dark, and a wire mesh floor. The mice were allowed to move freely through a tunnel between the two parts. The training trial was carried out after 4 days of the TMT injection. Mouse was placed in the light part and inescapable electric shock was provided (0.5 mA, 3 s) when the hind paws of the mouse were completely placed in the dark part. After single training trial, the passive avoidance test (5 days after TMT injection) was conducted. The mouse was again placed in the light part, and the time latency was measured and when consumed the mouse was reentered intothe dark part and step-through latency time into the dark part was evaluated (the step-through latency maximum testing limit was 300 s) [17].

The Morris water maze (MWM) test was carried out by referring to the Morris study with some modification [18]. The equipment consisted of a stainless steel circular pool (90 cm in diameter and 60 cm in height) that was randomly divided into quadrants (E, W, S, and N zones) with visual cues on the walls for navigation. The circular pool was filled up to 30 cm (high) using squid ink (Cebesa, Valencia, Spain) in water (22 ± 2°C). A platform (6 cm in diameter) was installed in the middle of the W zone, the position of which was unchanged during the training session. The mice were allowed to swim and the latency time until they escaped from the water onto the submerged platform up to a maximum of 60 s was recorded, and they were allowed to stay on the platform for 15 s. In the training session (6 days after TMT injection), the mice were subjected to swim for escape during four trials per day. The probe test (10 days after TMT injection) was conducted to evaluate the spatial memory and long-term memory without the platform for 60 s, and the time spent in the W zone was recorded using a SMART 3.0 video-tracking system.

### 2.8. Biochemical Analysis of the Mice Brains

For biochemical analysis, preparation for SOD activity involves homogenizing small pieces of whole brain with 40 volumes of ice-cold PBS. To get the pellets, the homogenates were directly centrifuged at 400 g for 10 min at 4°C. The pellets in 5–10 volumes of ice-cold 1x Cell Extraction Buffer (10% SOD buffer, 200 *μ*M phenylmethane sulfonylfluoride, and 0.4% (v/v) Triton X-100 in distilled water) were incubated on ice for 30 min and centrifuged at 10,000 g for 10 min at 4°C to get the supernatant. The protein concentration was determined using the Quant-iT protein assay kit (Invitrogen, Carlsbad, CA, USA).

The preparation for the determination of GSH and oxidized glutathione dimer (GSSG) level involves homogenizing small pieces of whole brain with 20 volumes of 5% metaphosphoric acid and direct centrifugation at 14,000 g for 15 min at 4°C to get the supernatant. To determine GSSG, the supernatant was treated with 2 M 4-vinylpyridine and incubated for 1 h at room temperature. The measurements of SOD and GSH were carried out using commercial kits. The concentration of protein was determined using the Quant-iT protein assay kit (Invitrogen, Carlsbad, CA, USA).

Brains of mice were dissected and homogenized with PBS corresponding to the 10 volumes of whole brain tissues. To get the supernatant, the homogenates were centrifuged at 10,000 g for 60 s at 4°C. 160 *μ*L of each supernatant was mixed with 960 *μ*L of 1% (v/v) phosphoric acid followed by addition of 320 *μ*L 0.67% (v/v) thiobarbituric acid solution. The mixture was incubated at 95°C in water bath for 1 h. The reactant (colored complex) was centrifuged at 1,600 g for 10 min, and absorbance of supernatant was measured at 532 nm using tetramethoxypropane as a standard. Malondialdehyde (MDA) levels as a token of lipid peroxidation were expressed as nmole/mg protein [8].

### 2.9. Phenolic Compounds Analysis

Analysis for physiological phenolics in EFAA was performed using the 3200 QTRAP with a hybrid triple quadrupole-linear ion mass spectrometer (Applied Biosystems, Foster City, CA, USA), and C_18_ column (250 × 4.6 mm, 5.0 *μ*m, ProntoSIL, BISCHOFF Chromatography, Germany) was used. The eluent solvents were used to A (0.1% formic acid in distilled water) and B (0.1% formic acid in acetonitrile), and a gradient condition was applied as follows (min, %B): (0, 20), (20, 60), (30, 90). The flow rate was 0.5 mL/min with a 20 *μ*L injection volume, column oven temperature of 30°C, and all the analyses were carried out using a TurboIonSpray ionization source, and ESI-MS conditions were as follows: negative-ion mode, curtain gas (N_2_) 20 (arbitrary units), drying gas (N_2_) heated to 650°C, and a variety of collision energies.

Analysis for contents of phenolics in EFAA was performed using the high performance liquid chromatography (HPLC) with a photodiode array UV-Vis detector system (Shimadzu Corporation, Kyoto, Japan), and C_18_ column (250 × 4.6 mm, 5.0 *μ*m, ProntoSIL, BISCHOFF Chromatography, Germany) was used. The mobile phase was used to acetonitrile: 10 mM KH_2_PO_4_ (10 : 90, v/v), isocratic, and monitored for 30 min (wavelength 205 nm). The flow rate was 1.0 mL/min with a 10 *μ*L injection volume and column oven temperature of 30°C.

### 2.10. Statistical Analysis

All data were expressed as mean ± SD. Verification of each average value was subjected to analysis of variance (ANOVA) using the SAS software (version 9.1, SAS Institute, Cary, NC, USA). Duncan's new multiple range test was used to determine the difference of means, and *p* < 0.05 was considered to be statistically significant. 

## 3. Results and Discussion

### 3.1. Cellular AChE Inhibitory Effect of EFAA

Neurodegenerative disease is related to damage or to the death of the neuronal cells that generate ACh as a neurotransmitter, and it can be decreased by AChE [1]. Drugs for neurodegenerative diseases have been used to maintain high ACh levels, but reported side effects include gastrointestinal disturbances [19]. Hence, an AChE inhibitor has been demanded that is a safe natural product without side effects, and our study also examined the AChE inhibitory effect of EFAA as a natural plant source.

**Figure 1:**
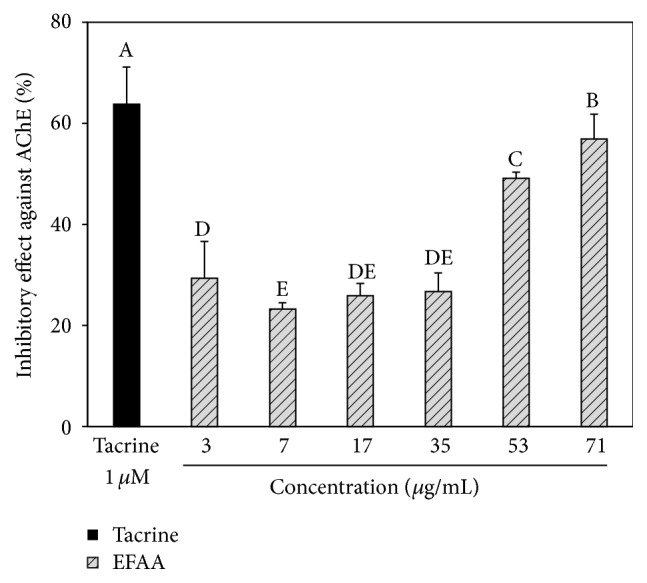
Inhibitory effect of ethyl acetate fraction from* Actinidia arguta* (EFAA) on cellular AChE. Inhibition was expressed as a percentage of enzyme activity inhibited with the control value (100%). Results shown are mean ± SD (*n* = 3). Data were statistically considered at *p* < 0.05, and different letters in graph represent statistical difference.

EFAA showed a significant AChE inhibitory effect similar to 1 *μ*M of tacrine (positive control) (Figure 1). Tacrine showed the highest inhibitory effect against AChE (63.79%), and most of the EFAA groups significantly inhibited the AChE activity and showed an IC_50_ value of 53 *μ*g/mL. Neurodegenerative diseases are related to reduced ACh levels as well as relatively high AChE levels resulting from the loss of cholinergic neurons [1]. The phenolics of a natural plant were reported to have an AChE inhibitory effect [3], and kiwifruit belonging to* Actinidia* spp. showed a high AChE inhibitory effect in* in vitro* analysis [20]. Therefore, the EFAA might be helpful in improving cognitive dysfunction through inhibition of AChE.

### 3.2. Inhibitory Effect of EFAA on Intracellular Oxidative Stress and Neuronal Cell Protective Effect of EFAA

Oxidative stress caused by excessive accumulation of ROS may impair neuronal cells, and this increased oxidative stress has been implicated in most neurodegenerative diseases [5]. Neuronal cells are particularly vulnerable to ROS, such as H_2_O_2_, and excessive exposure to ROS can lead to neurodegenerative diseases resulting from neuronal cell death [4]. Because cellular oxidative stress is an important factor in neurodegenerative diseases, such as AD, the effect of EFAA was measured by DCF-DA assay.

**Figure 2:**
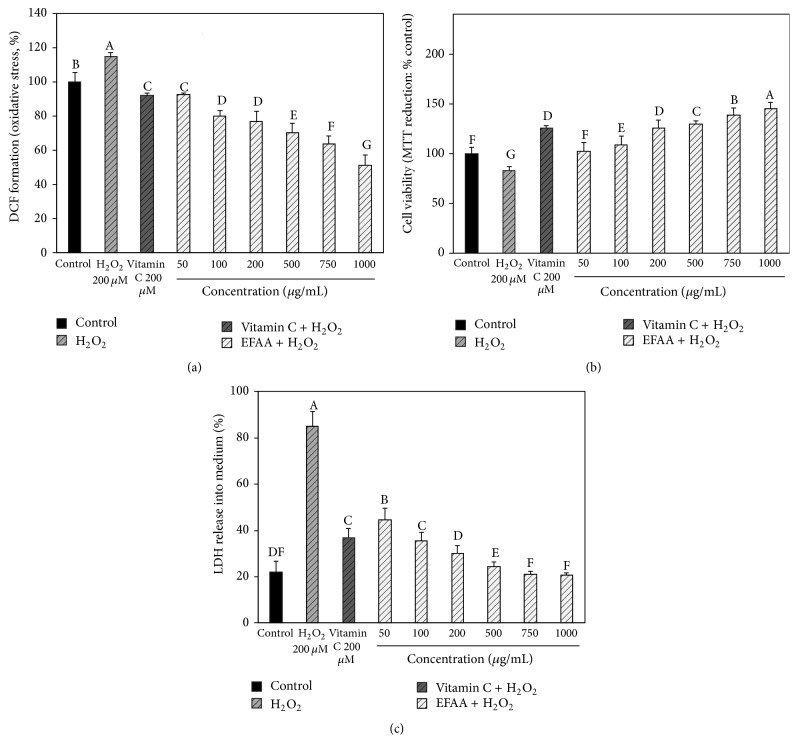
Protective effect of ethyl acetate fraction from* Actinidia arguta* (EFAA) on ROS production by the H_2_O_2_-induced cellular oxidative stress in PC 12 cells (a), neuronal cell protective effect on H_2_O_2_-induced cytotoxicity (b), and LDH release inhibitory effect on H_2_O_2_-induced membrane damage (c). Results shown are mean ± SD (*n* = 3). Data were statistically considered at *p* < 0.05, and different letters in graph represent statistical difference.

The H_2_O_2_ group intracellular oxidative stress level was increased (approximately 114.93%) compared with that of the control group (100.00%) (Figure 2(a)). In contrast, the EFAA groups' intracellular oxidative stress levels dose-dependently decreased compared with that of the H_2_O_2_ group. In particular, EFAA (1000 *μ*g/mL) showed a potent inhibitory effect (approximately 41%) on intracellular oxidative stress compared with the vitamin C group (92.16%), and all EFAA groups showed a significantly low oxidative stress level compared with that of the control group. These results indicated that EFAA protected the neuronal cells against H_2_O_2_-induced oxidative stress. A previous study reported that the* A. arguta* sprout showed a significant reducing effect on the intracellular ROS level caused by H_2_O_2_-induced oxidative stress [14]. In addition, natural antioxidants, such as phenolics, have a superior protective effect on neuronal cell damage caused by oxidative stress [6]. Therefore, phenolics in EFAA may protect neuronal cells by reducing increased oxidative stress.

Mitochondria might be regarded as one of the major targets that could be easily damaged by oxidative stress, causing neuronal cell death, because the induction of the mitochondrial permeability transition (MPT) pore can lead to mitochondrial cell death, which is related to the release of cytochrome C [21]. The cell viability of* A. arguta* on H_2_O_2_-induced neurotoxicity was examined by MTT assay, and the results are shown in Figure 2(b). The H_2_O_2_ group showed low cell viability (an approximately 17% decrease) compared with the control group (100.00%), and the neuronal cell protective effect (125.82%) of the vitamin C group was higher than in the control group. EFAA increased the cell viability compared with the H_2_O_2_ group and showed slightly higher cell viability than the vitamin C group in 500–1000 *μ*g/mL.

LDH is released into the medium by various ROS, which can lead to changes in the integrity and fluidity of the cell membrane, because neuronal cells have vulnerable structural characteristics against oxidative stress owing to a relatively high amount of lipid ingredients [4]. LDH was measured as a marker of neurodegenerative disease. The protective effect of EFAA against H_2_O_2_-induced cell membrane damage was examined by LDH assay, and the results are shown in Figure 2(c). The H_2_O_2_ group increased the LDH release quantity (approximately 60%) compared with the control group (22.07%), whereas the vitamin C group decreased the LDH release quantity (approximately 48%) compared with the H_2_O_2_ group, protecting the neuronal cell membranes. EFAA groups (≥200 *μ*g/mL) showed an excellent inhibitory effect on LDH release into the medium compared with the vitamin C group as a positive control. In particular, the EFAA groups (≥750 *μ*g/mL) showed a LDH release quantity similar to that of the control group.

Polyunsaturated fatty acids, such as linoleic acid and arachidonic acid, in the neuronal cells of the brain are weak to attacks by ROS [22]. Some studies have demonstrated significantly increased lipid peroxidation products (e.g., MDA, 4-hydroxynonenal, and acrolein) in the brains of AD patients [23]. If lipid peroxidation by ROS is inhibited, this may protect neuronal cells from acrolein, which expresses toxicity for the mitochondria [22]. The above results suggest that EFAA displayed protective effects on neuronal cells by inhibiting mitochondrial injury and cell membrane damage against H_2_O_2_-induced neurotoxicity.

### 3.3. Effect of EFAA on Behavioral Tests

Learning and memory impairments as the primary symptoms of AD have been related to the cholinergic system. TMT is known to cause various types of damage in terms of behavioral and biochemical deficits by causing pyramidal cell loss in the hippocampus as a potent neurotoxicant [11]. Therefore, our studies were carried out to confirm the beneficial effect of EFAA on TMT-induced cognitive dysfunction using Y-maze, passive avoidance, and MWM tests.

**Figure 3:**
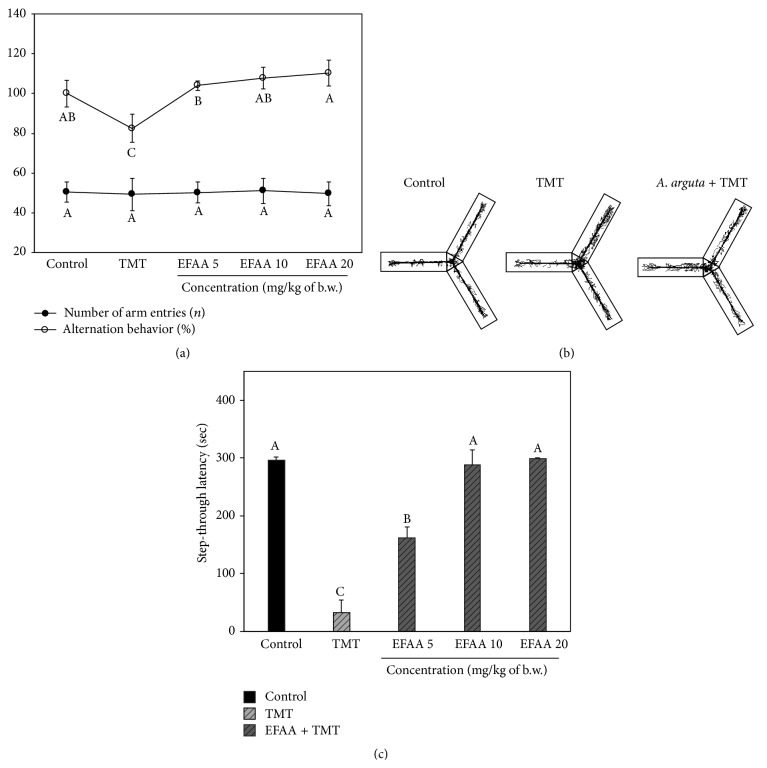
Protective effect of ethyl acetate fraction from* Actinidia arguta* (EFAA) against Y-maze and passive avoidance tests in TMT-induced amnesia. The spontaneous alternation behavior and number of arm entries (a) and path tracing of each group (b) were measured. Passive avoidance test was conducted 3 days after the TMT injection (c), and step-through latency (300 s) in the retention trial test was measured. Results shown are mean ± SD (*n* = 3). Data were statistically considered at *p* < 0.05, and different letters in graph represent statistical difference.

The Y-maze test was carried out using the innate tendencies of mice, which prefer to explore new environments in the maze rather than previously visited environments. In Figure 3(a), the TMT group showed impaired spatial working memories (118%, 18% decrease in alternation behavior) compared with those of the control group (100%). The EFAA groups showed increased alternation behavior (EFAA 5: 103.99%, EFAA 10: 107.65%, and EFAA 20: 110.21%). In contrast, the basic motor ability of the mice was not affected by TMT, because the number of arm entries showed no statistical difference between all experimental groups. Furthermore, the TMT group indicated that abnormal behavior, such as hyperactivity, was induced, because the movement routes of the TMT group were imbalanced compared with those of the control group. However, the EFAA group showed a similar shape to that of the control group (Figure 3(b)).

A passive avoidance test was performed to examine learning and short-term memory abilities in mice, which were given an unavoidable electronic shock when entering a dark place (Figure 3(c)). The TMT group showed a significantly low latency time (32.83 s, an 88.95% decrease in step-through latency) compared with that of the control group (297.20 ± 4.76 s). In contrast, the EFAA groups showed effectively attenuated step-through latency against TMT-induced impairment (EFAA 10: 288.20 s and EFAA 20: 298.75 s).

**Figure 4:**
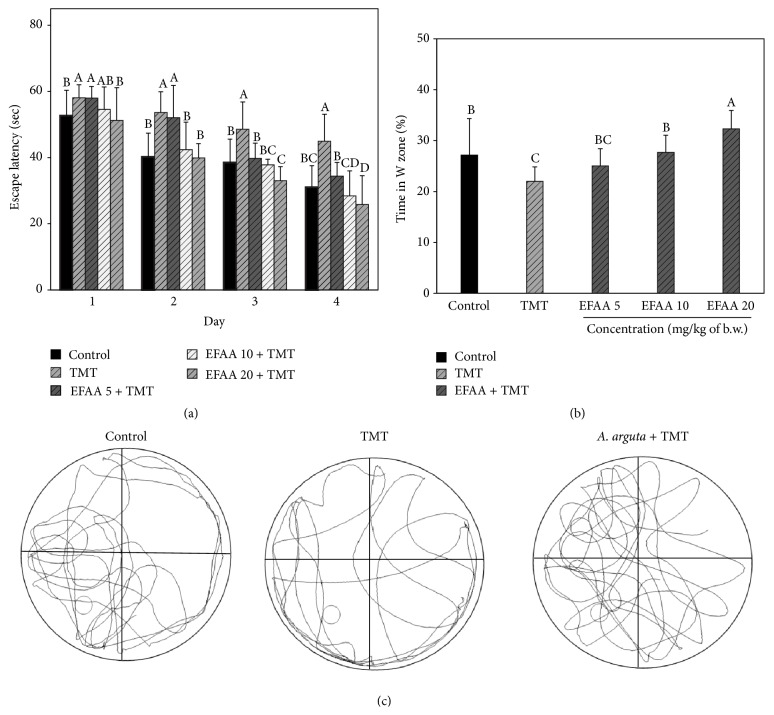
Protective effect of ethyl acetate fraction from* Actinidia arguta* (EFAA) against Morris water maze test in TMT-induced amnesia. The test was performed 5 days after the TMT injection, and escape latency in the training trial (a), platform crossings of probe trial sessions (b), and movement routes of each group in the probe trial (c) were measured during the 5 days. Results shown are mean ± SD (*n* = 3). Data were statistically considered at *p* < 0.05, and different letters in graph represent statistical difference.

Based on these results, a long-term learning and spatial memory test was performed via the MWM test, and the results are shown in Figure 4(a). The TMT groups showed relatively high escape latency times on days 1, 2, 3, and 4 during the training trials compared with the control group. However, the EFAA groups showed decreased escape latency time during the training trials compared with the TMT group. In particular, the EFAA 20 group had lower escape latency times on days 3 and 4 in the training trials than the control group. After the training trials, long-term learning and spatial memory were examined in the probe trial without the platform. The TMT group spent relatively less time (22.00%) in the W zone compared with the control group (27.15%); however, the EFAA groups showed a relatively higher retention time (EFAA 5: 25.05%, EFAA 10: 27.69%, and EFAA 20: 32.31%) in the W zone (Figure 4(b)). In addition, Figure 4(c) shows the movement routes of the mice in each group. The EFAA groups showed a relatively large amount of movement routes in the platform area compared with the other areas, whereas the TMT group showed random moving patterns. The mice of the EFAA group spent more time in the platform area compared with the TMT group.

The brains of rodents, including the hippocampus and prefrontal cortex, are involved in tasks such as learning and memory [18]. Previous research has reported that TMT exposure may lead to neurodegenerative disease, including neurobehavioral alteration, behavioral abnormality, aggression, and learning impairment caused by hippocampal damage [11]. Therefore, the present* in vivo* results demonstrated TMT-induced hippocampal damage via behavioral tests (Y-maze, passive avoidance, and MWM tests) of mice, and the ameliorating effect of EFAA was confirmed. According to previous studies, ferulic acids as phenolics showed an ameliorating effect on cognitive function against TMT-induced amnesia in* in vivo* tests (Y-maze and passive avoidance tests) and phenolics protected neuronal cells from damage by oxidative stress and increased ChAT activity [2]. In our results, the TMT-exposed mice showed low cognitive ability in each behavioral test compared with the control group, and this cognitive dysfunction caused by TMT was consistent with the findings of a previous study [12]. The EFAA groups showed improved learning and memory functions against TMT-induced cognitive deficit, and these beneficial effects may be considered as affected by phenolics in EFAA. Additionally, hippocampus lesions are related to spatial memory impairment [18]. The TMT group may have experienced significant hippocampal damage caused by TMT, because the TMT-exposed mice showed decreased escape ability in the training trials and probe tests in the MWM test. In addition, the EFAA group showed improved long-term and spatial memory, owing to relatively much more time spent in the platform area than that of the TMT-exposure mice. These results strongly suggest that phenolics in EFAA may have a significant effect on improving cognitive function. Therefore, phenolics in EFAA not only might be helpful for improving cognitive function by protecting neuronal cells or inhibiting AChE but also could enhance spatial memory and long-term memory against TMT-induced amnesia.

### 3.4. AChE Activity in Mice Brain Tissues

**Figure 5:**
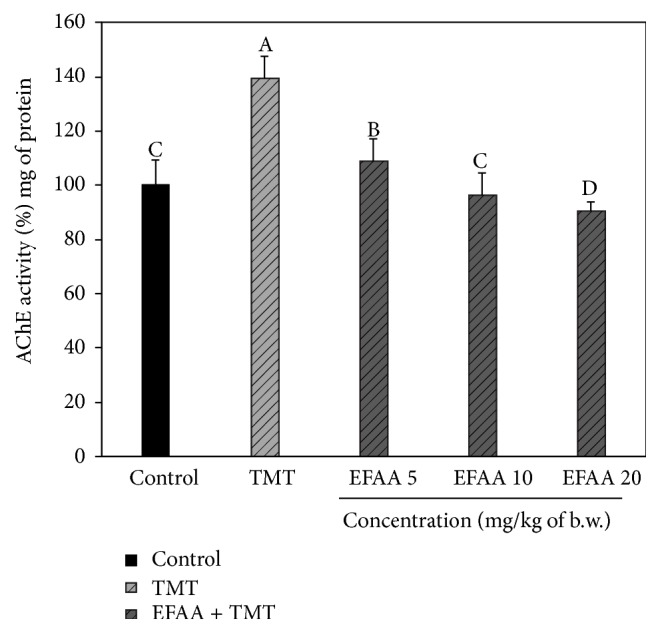
Inhibitory effect of ethyl acetate fraction from* Actinidia arguta* (EFAA) on AChE from TMT-induced defective mice brain homogenates. Results shown are mean ± SD (*n* = 3). Data were statistically considered at *p* < 0.05, and different letters in graph represent statistical difference.

Regarding the mechanisms of TMT-induced amnesia, it has been speculated that damage to the cholinergic system in the hippocampus is related to the change of neurotransmitters, as well as neuronal cell loss. ACh level plays an important role in the modulation of cognitive performance and signal transfer in the synapses, and it may correlate with neurodegenerative disease [24]. Therefore, TMT-induced AChE activation in mice brains and the inhibitory effect of EFAA were investigated. In Figure 5, the TMT group shows increased AChE activity (approximately 39%) compared with the control group (100.00%), whereas the AChE activity of the EFAA groups is significantly decreased. In particular, the EFAA 20 group showed decreased AChE activity (approximately 10%) compared with the control group. According to a previous study, the brain tissues of TMT-induced ICR mice showed high AChE activity and* Poncirus trifoliata* extract significantly inhibited AChE activity [12]. This was consistent with our results, which show increased AChE activity by TMT, and EFAA displayed inhibitory activity against TMT-induced AChE in the brain tissues. Excessive release of AChE, which is well known as a biomarker for memory malfunction, might be a factor in reducing cognitive function owing to the acceleration of the hydrolysis of ACh [3]. The present study suggests that EFAA is an effective natural source against AChE inhibition, because EFAA has a strong effect on ameliorating cognitive impairment caused by AChE overactivation.

### 3.5. Biochemical Analysis of Mice Brain Tissues

**Figure 6:**
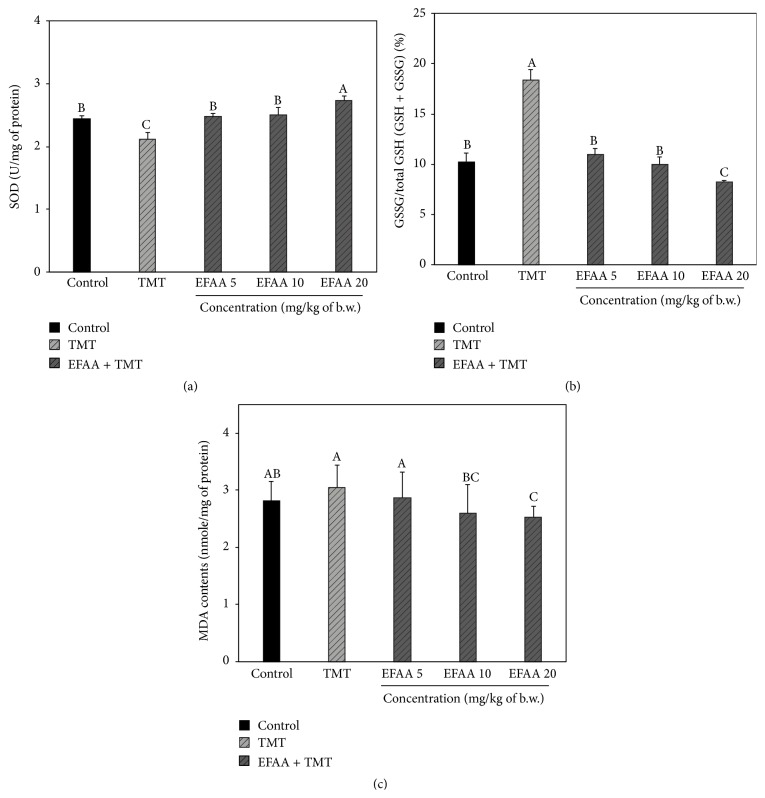
Effect of ethyl acetate fraction from* Actinidia arguta* (EFAA) on SOD contents (a), GSSG/total GSH (GSH + GSSG) ratio (b), and MDA contents (c) from TMT-induced defective mice brain homogenates. Results shown are mean ± SD (*n* = 3). Data were statistically considered at *p* < 0.05, and different letters in graph represent statistical difference.

SOD, which is one of the antioxidant enzymes, is an enzyme that catalyzes the conversion of superoxide radical into molecular oxygen (O_2_) and H_2_O_2_. H_2_O_2_ is sequentially neutralized through scavenging by catalase, peroxidase, and variable antioxidants. SOD has a role in maintaining physiological redox balance or reducing oxidative stress [25]. A previous report showed that SOD activity in brain tissue was diminished by TMT exposure in mice [26]. In Figure 6(a), the TMT group shows SOD activity (2.19 U/mg protein) and the EFAA 20 group shows statistically increased SOD activity (2.72 U/mg protein) compared with the TMT group.

The GSH of the brain tissues is also involved in the detoxification process of intracellular ROS, including free radicals, lipid peroxides, and electrophilic substances [25]. Irreversible damage occurs when the neuronal cells are not able to maintain GSH homeostasis, and the deficiency of GSH accelerates the signal processes leading to TMT-induced neuronal cell death [27]. In contrast, phenolics may help to maintain the homeostasis of GSSG/GSH owing to their antioxidant activity [28]. The ratio of GSSG/GSH is often used to indicate the oxidative stress level in the cell. In Figure 6(b), the TMT groups show an increased GSSG/total GSH (GSH + GSSG) ratio (approximately 18%) compared with the control group (10.22%). However, the EFAA groups show a decrease in the GSSG/total GSH (GSH + GSSG) ratio. The EFAA 20 group presents a lower GSSG/total GSH (GSH + GSSG) ratio (8.20%) than the control group.

MDA, the final product of lipid peroxidation generated in damaged tissues by ROS, such as free radicals, is considered an indicator of oxidative stress [26]. Since the brain tissue has more plentiful unsaturated fatty acids than other tissues, oxidative stress occurring in the brain tissue continuously leads to memory loss and cognitive disorders [23]. TMT exposure has been found to increase the production of MDA through an excitotoxic effect on neuronal cells in the hippocampus of the brain [26]. Therefore, the lipid peroxidation levels of TMT-induced amnesia were estimated by the amount of MDA in brain tissues. The TMT group showed slightly higher MDA contents (3.04 nmole/mg protein) than the control group (2.82 nmole/mg protein), while EFAA was shown to inhibit lipid peroxidation in mice brain tissues (Figure 6(c)). The EFAA 20 group showed effectively inhibited lipid peroxidation (approximately 18%) compared with the TMT group.

In our results, EFAA was shown to inhibit the biochemical change against TMT-induced oxidative stress in the mice brain tissues. Previous research reported that* A. arguta* has various phenolics, including chlorogenic acid, catechin, rutin, and quercetin [7]. Chlorogenic acid has also been reported to increase the GSH level by scavenging the streptozotocin-induced oxidative stress in diabetes [29]. SOD and GSH are the most important enzymes in the cell antioxidant defense system, and their quantitative balance could inhibit the MDA production resulting from lipid peroxidation. Therefore, EFAA could assist the improvement of TMT-induced amnesia by increasing SOD activity and GSH activity and inhibiting the production of MDA.

### 3.6. Identification of Main Phenolics in EFAA

**Figure 7:**
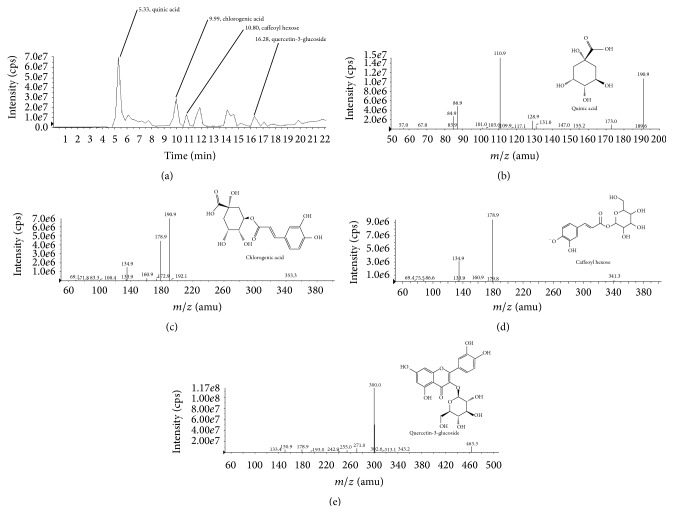
Liquid chromatography coupled with 3200 QTRAP mass spectrometer in negative-ion mode. Base peak chromatogram of mass scan (a), MS^2^ scan data for quinic acid (b), chlorogenic acid (c), caffeoyl hexose (d), and quercetin-3-glucoside (e). MS^2^ data patterns indicated a variety of collision energies (CE) −20 eV (b), −29 eV (c), −28 eV (d), and −35 eV (e), respectively.

The phenolics of EFAA were identified by 3200 QTRAP with a hybrid triple quadrupole-linear ion trap mass spectrometer for retention time, UV-Vis spectrum, a full scan of mass data, and an MS^2^ scan for mass fragmentation. Four phenolics in EFAA were identified as two major peaks (retention time at 5.33 and 9.99 min) and two minor peaks (retention time at 10.80 and 16.28 min) by the base peak chromatogram of mass scan (Figure 7(a)). The main four peaks presented in the ESI-MS spectra were identified by the molecular ions [M-H]^−^ at* m/z* values of 190.9, 341.3, 353.5, and 463.3, respectively. These four molecular ions were tentatively identified by a detailed analysis of their negative MS^2^ scan data. The product ion at* m/z* 190.9 was observed to have fragment ions at* m/z* 173.0, 128.9, 110.9, 101.0, 86.9, and 84.9 (Figure 7(b)); these data were consistent with the fragment ions of quinic acid [30]. The product ion at* m/z* 353.5 (Figure 7(c)) was tentatively identified as a 3-*O*-caffeoylquinic acid (chlorogenic acid) that has fragment ions at* m/z* 190.9, 178.9, 172.9, and 134.9 via LC/MS^3^ data analysis for monoacylchlorogenic acid [30, 31]. In Figure 7(d), caffeoyl hexose as a deprotonated molecular ion at* m/z* 341.3, which coupled with a dehydrated hexose at* m/z* 178.9 and decarboxylated caffeoyl at* m/z* 134.9, was also identified [30]. Finally, the product ion at* m/z* 463.3 (Figure 7(e)) was identified as a quercetin-3-glucoside through comparison with the MS^2^ fragment ions reported previously, which showed the loss of a dehydrated glucoside at* m/z* 300.0, and the fragment ions were confirmed as follows: at* m/z* 300.0, 271.0, 255.0, 178.9, and 150.9 [32].

*A. arguta* is widely known as a food with antioxidant properties, and it is steadily consumed. Due to the rich phenolics in* A. arguta*, it has been studied in terms of its antioxidant, anti-inflammation, antidiabetic, antiallergic, and other effects [13, 14]. A previous study investigated the polyphenol contents of kiwifruit (*A. deliciosa*), and various phenolic compounds were found, including caffeic acid, ferulic acid, syringic acid, ellagic acid, quercetin, catechol, pyrogallol, vanillin, and gallic acid [33]. Although the main phenolics in EFAA were confirmed as different compounds to* A. deliciosa*, these phenolics may be considered as having an ameliorating effect on cognitive function. Chlorogenic acid is one of the caffeoylquinic acid derivatives, including chlorogenic acid, 1,3-di-*O*-caffeoylquinic acid, and 1,5-di-*O*-caffeoylquinic acid. It is an ester formed between quinic acid and caffeic acid, and it is a polyphenol widely present in the leaves and fruits. It has also been reported to have good antioxidant activity, as evidenced by the decrease in oxidative stress [29]. Another report stated that although the antioxidant mechanisms of chlorogenic acid are still unclear, the antioxidant ability of chlorogenic acid is expected because of its redox-regulated transcription factors [28]. In addition, the effects of chlorogenic acid were also reported to ameliorate scopolamine-induced amnesia by inhibiting AChE, oxidative stress, and lipid peroxidation [34]. Caffeoyl hexose, which belongs to the hydroxycinnamic acid family, is collectively known as chlorogenic acid. Additionally, quinic acid is a colorless crystalline acid obtained from plant products or made synthetically by the hydrolysis of chlorogenic acid. Caffeoyl hexose and quinic acid have not yet been researched in many studies, but they may be helpful as antioxidants by scavenging the free radical. Finally, quercetin is one of the flavonoids that has potent antioxidant properties compared with vitamin C and vitamin E, and it was reported to protect the neuronal cell against A*β*_1−42_-induced neurotoxicity [35]. Since recent research reported that* A. arguta* contains the quercetin-3-glucoside compound, our study also tentatively identified quercetin-3-glucoside among various quercetin derivatives [13]. In addition, the free radical scavenging activity of quercetin-3-glucoside was reported to show higher activity than phloridzin, 3-hydroxyphloridzin, chlorogenic acid, epicatechin, epicatechin dimer (procyanidin B2), trimer, and quercetin-3-glucoside [36]. Quinic acid, as a main phenolic compound in EFAA, was analyzed as 32.10 *μ*g/mg of* A. arguta*.

Consequently, EFAA, including four phenolics, showed a significant antiamnesic effect on TMT-induced cognitive defects through AChE inhibition and antioxidant activity in a mouse model. Therefore, in this assay, the identified phenolics, including chlorogenic acid and quercetin-3-glucoside, were found to potentially contribute to the enhancement of cognitive function. Therefore, it could be considered that the learning and memory effect of EFAA may be due in part to the presence of chlorogenic acid, quercetin-3-glucoside, quinic acid, and caffeoyl hexose.

## 4. Conclusion

EFAA showed significant cellular AChE inhibitory effect and neuronal cell protective effect based on the cellular antioxidant activity caused by the H_2_O_2_. TMT-induced amnesia in the ICR mouse model was effectively improved by EFAA treatment. After* in vivo* behavioral tests, mice brain tissues were collected for examining AChE activity and several antioxidant systems. EFAA showed AChE inhibitory effect in brain and excellent antioxidant activity in SOD, GSSG/total GSH (GSH + GSSG), and MDA assay. The ameliorating effect of EFAA on TMT-induced amnesia may be affected by its main phenolics identified as a quinic acid, chlorogenic acid, caffeoyl hexose, and quercetin-3-glucoside. Consequently, our results suggest that* A. arguta* as natural food resources might be considered possible substance to prevent neurodegeneration through AChE inhibition and strong antioxidant activity.
